# Guide to the Clinical Examination and Treatment of Sick Children

**Published:** 1899-06

**Authors:** 


**Guide to the Clinical Examination and Treatment of Sick Children.** By John Thomson, M.D. Pp. xvi., 336. Edinburgh: William F. Clay. 1898.

Dr. Thomson's work is a useful manual for students, but, as he says, it is only supplementary to larger works on the same subject. While he was engaged in the work it seems a pity a more complete guide to clinical examination of children was not produced, as at present some parts might well have been amplified with advantage, more particularly where any help is intended to be given on treatment. The illustrations are anything but a success, and we very much doubt whether anyone could gain much from Fig. 8 or 9, or several others. Diagnosis at sight may be a useful faculty to possess, but it is a dangerous one on which to rely. We hope in a future edition to see these photographs left out, or very materially improved.

